# Taming the model zoo: a unified view on correlations in recurrent networks

**DOI:** 10.1186/1471-2202-13-S1-P147

**Published:** 2012-07-16

**Authors:** Dmytro Grytskyy, Moritz Helias, Tom Tetzlaff, Markus Diesmann

**Affiliations:** 1 Institute of Neuroscience and Medicine (INM-6), Computational and Systems Neuroscience, Research Center Jülich, Germany; 2 Faculty of Medicine, RWTH Aachen University, Germany; 3 RIKEN Brain Science Institute, Wako City, Japan

## 

The meaning of correlated neural activity for the processing and representation of information in cortical networks is still not understood, but evidence for a pivotal role of correlations increases [1]. Recent theoretical work has shown [2-4] that balanced recurrent networks of binary model neurons [3] and spiking integrate-and-fire models [2-4] are able to produce weak correlations despite common input to pairs of cells. For binary model neurons, the theory of correlations in recurrent networks is well established [5]. Investigating learning in recurrent networks with spike-timing dependent plasticity requires spiking neuron models. Theoretical work often employs linear stochastic point process models [6] for their analytic tractability [7]. The diversity of neuron models used in contemporary theoretical neuroscience brings up the question, which features of correlations are generic properties of recurrent networks and which are peculiarities of the often abstracted neuronal dynamics. Moreover, the variety of different theories employed to describe pairwise correlations in neural networks is confusing at times, even for experts in the field. Currently it is unclear how different neuron models relate to each other and whether and how results obtained with one model carry over to another. In this work we present a unified theoretical view on pairwise correlations in recurrent random networks. We consider binary neuron models, leaky integrate-and-fire models, and linear point process models. For networks in the asynchronous irregular regime, we show that these models can be mapped to either of two definitions of an Ornstein-Uhlenbeck (OU) process [8]. The distinction between both classes is how the effective noise enters the model: Leaky integrate-and-fire models and spiking point process models belong to the class with noise on the output side, the binary neuron model is equivalent to an OU process with noise on the input side. The closed solution for the correlation structure of OU processes [8] holds for both classes. We extend this solution to the presence of synaptic conduction delays. The presented theory recovers and unifies the theories of correlations for binary neurons [5] and linear point processes [7] and generalizes both models to the case of finite conduction delays. Moreover we obtain a good approximation for the temporal structure of correlations for the spiking leaky integrate-and-fire model in the asynchronous regime [9]. Finally we show that the oscillatory instability known for networks of integrate-and-fire models [9] is a model-invariant feature of any of the studied dynamics and we explain the class dependent differences in the temporal shape of correlation functions.
